# Reduced Awareness for Osteoporosis in Hip Fracture Patients Compared to Elderly Patients Undergoing Elective Hip Replacement

**DOI:** 10.3390/medicina58111564

**Published:** 2022-10-31

**Authors:** Moritz Kraus, Carl Neuerburg, Nicole Thomasser, Ulla Cordula Stumpf, Matthias Blaschke, Werner Plötz, Maximilian Michael Saller, Wolfgang Böcker, Alexander Martin Keppler

**Affiliations:** 1 Department of Orthopaedics and Trauma Surgery, Musculoskeletal University Center Munich, Ludwig-Maximilians University Munich, Marchioninistr. 15, 81377 Munich, Germany; 2 Department of Gastroenterology, University Hospital Augsburg, Stenglinstraße 2, 86156 Augsburg, Germany; 3 Hospital Barmherzigen Brüder München, Teaching Hospital Technial University Munich, Romanstraße 93, 80639 Munich, Germany

**Keywords:** awareness, osteoporosis, proximal femur fracture, hip replacement, fracture liaison service

## Abstract

*Background:* Osteoporotic fractures are associated with a loss of quality of life, but only few patients receive an appropriate therapy. Therefore, the present study aims to investigate the awareness of musculoskeletal patients to participate in osteoporosis assessment and to evaluate whether there are significant differences between acute care patients treated for major fractures of the hip compared to elective patients treated for hip joint replacement.; *Methods:* From May 2015 to December 2016 patients who were undergoing surgical treatment for proximal femur fracture or total hip replacement due to osteoarthritis and were at risk for an underlying osteoporosis (female > 60 and male > 70 years) were included in the study and asked to complete a questionnaire assessing the awareness for an underlying osteoporosis. ASA Score, FRAX Score, and demographic information have also been examined. *Results:* In total 268 patients (female = 194 (72.0%)/male = 74 (28%)), mean age 77.7 years (±7.7) undergoing hip surgery were included. Of these, 118 were treated for fracture-related etiology and 150 underwent total hip arthroplasty in an elective care setting. Patients were interviewed about their need for osteoporosis examination during hospitalization. Overall, 76 of 150 patients receiving elective care (50.7%) considered that an examination was necessary, whereas in proximal femur fracture patients the awareness was lower, and the disease osteoporosis was assessed as threatening by significantly fewer newly fractured patients. By comparison, patients undergoing trauma surgery had a considerably greater risk of developing another osteoporotic fracture than patients undergoing elective surgery determined by the FRAX^®^ Score (*p* ≤ 0.001).; *Conclusions:* The patients’ motivation to endure additional osteoporosis diagnostic testing is notoriously low and needs to be increased. Patients who underwent acute care surgery for a fragility proximal femur fracture, although acutely affected by the potential consequences of underlying osteoporosis, showed lower awareness than the elective comparison population that was also on average 6.1 years younger. Although elective patients were younger and at a lower risk, they seemed to be much more willing to undergo further osteoporosis assessment. In order to better identify and care for patients at risk, interventions such as effective screening, early initiation of osteoporosis therapy in the inpatient setting and a fracture liaison service are important measures.

## 1. Introduction

The importance of bone quality in the proximal femur is paramount, as it can have a significant hazard for fragility fractures and consequent proper implantation of hip prostheses. The treatment of femoral neck fractures is one of the most frequently performed procedures worldwide. Due to the changing demographics, the number of proximal femur fractures (PFF), one of the most frequent fractures in older people, is predicted to rise to 6.3 million per year by 2050 [1]. Between 2020 and 2040, the number of hip fracture patients who experience a decline in health as measured by DALYs (disability adjusted life years) is expected to double, while socioeconomic expenses are anticipated to rise by 65% [2]. Along with a loss in mobility and daily activities, the mortality rises by up to 20% in the first year [3,4]. Even though osteoporosis following PFF is highly prevalent, the percentage of proper treatment is appallingly low, particularly in Germany [5]. Furthermore, affected patients have very low awareness of the disease osteoporosis [6].

Patient awareness of osteoporosis management is also likely to have a relevant impact on secondary fracture prevention. Considering the known treatment gap in patients with osteoporosis and taking into account the growing awareness of osteoporosis among surgeons, the purpose of this study was to ascertain the willingness of elective and non-elective patients undergoing hip surgery to participate in further investigations and subsequent treatment of osteoporosis.

Patients undergoing hip replacement due to osteoarthritis were used as an elective surgery comparison group (ES) to the patients undergoing non-elective surgery (NES) due to hip fracture. Although patients with a planned total hip arthroplasty often have risk factors for osteoporosis, no regular assessment is performed [7]. This is all the more important because, in addition to the femoral neck, regions such as the distal radius or the spine also have an increased fracture risk in patients suffering osteoporosis. It has been shown that underestimating the prevalence of osteoporosis may increase the perioperative fracture risk. Osteologists and arthroplasty surgeons must be cautious of postoperative alterations in bone density. In fact, it is advised to take into account regular bone mineral density (BMD) screening after knee arthroplasty [8]. It has been shown that occult osteoporosis is present in 25% of patients with hip osteoarthritis and thus osteoporosis screening is quite reasonable in this cohort [9]. In addition to osteoarthritis, osteoporosis patients often have comorbidities with other diseases such as hypertension, diabetes mellitus and depression [10,11,12]. The COVID-19 pandemic of recent years has also had an impact, particularly on patients with proximal femur fracture. An Italian multicenter study found a decrease in the mean age of patients with proximal femur fracture and a higher incidence of domestic falls than before the pandemic [13].

The purpose of this study was to evaluate differences in fracture risk, demography, and subjective health status among the groups listed in terms of awareness of and risk factors for osteoporosis.

## 2. Materials and Methods

All patients (female > 60 years or male > 70 years) who received elective total hip arthroplasty or hip fracture surgery in the trauma surgery department of a maximum care university hospital or in an orthopedic clinic in the same city between May 2015 and December 2016 were included in the study. The most significant risk factors for osteoporosis (according to Dachverband für Osteologie e.V. (DVO)) [14] and the patients’ general willingness to undergo osteoporosis diagnostics and other screening programs provided by health insurance companies (mammography and coloscopy) were assessed by a questionnaire to determine the patients’ awareness of osteoporosis. This questionnaire was already published in a previous paper by our working group [6]. In addition, the 10-year fracture risk for hip fracture and other fragility fractures was determined in all patients using the WHO Fracture Risk Assessment Tool (FRAX^®^). Patients with the following conditions were disqualified: language barriers, patients with further fractures, and patients with brain organic illnesses (such as dementia, delirium, etc.). The Local Ethics Committee of the University gave its approval to the project. (AZ 351-14).

### 2.1. Questionnaire

To assess awareness of osteoporosis, readiness for osteoporosis diagnosis, and risk factors according to DVO and FRAX^®^, the questionnaire was specifically designed. It includes information on age, height, and weight as well as a self-report of current health state using numerical and visual analogue rating scales. This has already been used by our research group to study awareness and risk factors in osteoporosis patients with distal radius fracture [6].

### 2.2. Self-Assessment of Health Status by Rating Scale

To evaluate a state in relation to a certain trait, rating scales were utilized. Patients can characterize their present state of health using the one-dimensional numerical rating scale in the questionnaire. The WHO defined health as a condition of whole mental, bodily, and social well-being in 1947. Patients were asked to mark a rating scale level on a scale from 0 to 10, where 10 represents major disease (=severely ill) and 0 represents total health. A smiley-based symbolic rating system was employed to make it easier for elderly patients who no longer possess the essential ability to abstract to utilize a numerical rating scale. Additionally, the American Society of Anesthesiologists (ASA) score, which was gathered by associates in the anesthesia department before to surgery, was used to evaluate the patients’ physical health status.

Further questions regarding the patient’s health status, information, and awareness of osteoporosis were created in a binary nominal scale as “yes/no” responses to help with response and evaluation. As a result, details about osteoporosis that was known or diagnosed, such as current treatment, were elicited. Likewise, participants were questioned regarding their own subjective need for an osteoporosis screening and their perception of the disease’s risk. Other preventive exams, including colonoscopies for both men and women within the last ten years, routine prostate exams for men, and mammography screening for women up to 70 years of age were all noted.

According to DVO guideline 2014 [14], risk variables for osteoporosis were gathered in order to ascertain each individual’s 10-year risk of fractures and the justification for general and/or targeted medication therapy for osteoporosis. The following characteristics were covered: wrist, vertebral, and femoral fractures that developed after the age of 50, parental hip fractures, nicotine use, glucocorticoid use, and underlying illnesses, such as type 1 diabetes, rheumatoid arthritis, osteogenesis imperfecta, untreated hyperthyroidism, hypergonadism, chronic liver disease, malnutrition, alcohol use (less than one bottle of beer or glass of wine per day), daily dairy intake, and regular vitamin D intake. Menopause before the age of 45 in women, as well as consistent usage of aromatase inhibitors.

### 2.3. Fracture Risk Assessment Tool (FRAX^®^)

The Fracture Risk Assessment Tool (FRAX^®^) is a fracture risk assessment tool published in 2008 by the World Health Organization (WHO) Collaborating Centre for Metabolic Bone Diseases at Sheffield, UK. It provides country-specific algorithms for estimating the individual 10-year probability of hip fracture and other serious osteoporotic fractures of the pelvis, vertebral bodies, distal radius, and proximal humerus. The calculation of fracture probability is based on the factors: age, gender, height, weight, previous fracture, parental hip fracture, smoking status, oral glucocorticoid use, rheumatoid arthritis, secondary osteoporosis, alcohol intake and bone mineral density at the femoral neck. The algorithm queries these risk factors, which, depending on age and BMI, and electively bone density, results in a percentage 10-year fracture risk. This can be determined either in the form of cross-tabulations or using the FRAX-online calculator. The probability obtained in this way can be used to decide on therapy options. Kanis et al. give age-related threshold values for the classification into risk groups, which can be considered as an indication for specific osteoporosis therapy [15].

### 2.4. Osteoporosis Screening

The assessment of respondents’ physical activity, fall risk, dietary practices, and medication use also helped to estimate each person’s individual risk for osteoporotic fractures. Our treatment strategy, which is in line with our osteoporosis guidelines, also included basic osteoporosis laboratory tests and spine radiographs as needed to detect common vertebral fractures. [16].

### 2.5. Statistical Analysis

Subjects were classified into four age groups: Group G1 = 60–69 years, G2 = 70–79, G3 = 80–89, and G4 = 90–99 years. First, the relationship between patients’ age and awareness of osteoporosis was determined and analyzed descriptively. Data are presented as mean ± standard deviation (SD). The prevalence of risk factors for osteoporosis was determined bivariate using cross-tabulations and tested for significance with a chi-square test. An unpaired t-test with a significance level of *p* ≤ 0.05 was performed to compare the influencing factors. To test for a linear relationship between ordinal scaled variables, a correlation analysis using Spearman-Rho was performed.

Normal distribution and comparability of variance of the data were tested using the Shapiro–Wilk and Levene’s test. To compare two interval scaled normally distributed samples with comparable variance, the unpaired students t-test was used. The Mann–Whitney U test and the 2-sample test for equality of proportions with continuity correction were used to examine if the central tendency of two independent samples differed.

In addition, multiple logistic regression was performed using the “glm” function of the “stats” package (version 3.6.2) for the factors influencing outcome awareness, including the parameters age, sex, BMI, ASA score, ethnicity, number of risk factors, and previous fracture.

R software version 4.0.3 was used to conduct statistical analysis (R Foundation for Statistical Computing, Vienna, Austria). The same software’s package ggplot2 version 3.3.2 was used to make each graph.

## 3. Results

In total, 268 patients with an average age of 77.7 years (±7.7), (female = 194 (72%)/male = 74 (28%)) were included in the study of which 150 patients (56%) were treated electively and 118 patients (44%) were treated non-electively. Patient characteristics including BMI, age group distribution, ASA scores, proportions of risk factors for osteoporosis and patient reported health status as displayed in Table 1.

### 3.1. Self-Assessment of Health Using a Rating Scale and a Questionnaire

The NES group reported an average score of 4.46 ± 2.42 as their subjective health state at the time of the interview, using a numerical rating scale in which 0 represents perfect health and 10 represents severe disease. For ES patients, a significantly lower mean score of 3.87 ± 2.42 (*p* < 0.0001) was recorded.

#### Body Mass Index

The mean body mass index (BMI) was 24.05 ± 4.27 kg/m^2^ in the NES patients. The patients in the ES group had a significantly higher BMI with an average of 25.12 ± 3.94 kg/m^2^ (*p* = 0.031). The prevalence of obesity classes as defined by the World Health Organization among the two groups is shown in Table 1.

### 3.2. ASA-Score

The ASA score was higher in the NES group, 2.61 ± 0.58 points, compared with an average of 2.05 ± 0.49 points in the ES group. It should be noted here that mean age in the two groups is significantly different (*p* = 0.020). ES patients were 75.0(±6.40) years old, whereas NES patients were on average years old 81.25 (±7.80).

### 3.3. Risk Factors and FRAX

On average, patients in the NES group had slightly more risk factors (1.86 ± 1.45) based on the DVO guidelines than the ES group (1.25 ± 1.12). The prevalence of the queried risk factors for osteoporosis is shown in Table 1. According to FRAX^®^-Score, “high risk” is defined as having a 10-year fracture hazard of ≥3% for hip and/or ≥20% for other major osteoporotic fractures, while “low risk” refers to risk percentages that are lower than these cutoff points. Of the investigated NES patients, 35% (41/118) had a 20% chance of suffering a severe osteoporotic fracture in the following ten years. In contrast, the proportion in the ES group was only 25% (38/150). The proportion at high risk of hip fracture within the next 10 years was significantly lower in the ES group at 70% (105/150) than in the NES group at 92% (108/118), (*p* ≤ 0.0001).

Only 46.6% (55/118) of NES patients and 50.7% (75/150) ES patients considered further diagnostics regarding osteoporosis necessary. This was significantly lower than the proportion of patients who underwent a precautionary colonoscopy (NES: 62% vs. ES: 66%), or regularly underwent general preventive examinations by the health insurers (NES: 67% vs. ES: 85%).

**Figure 1:**
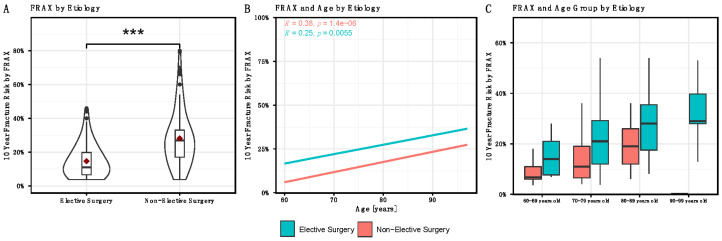
(**A**): FRAX^®^ Score by Aetiology: (**A**): Distribution of FRAX-Score by Aetiology. FRAX score was found to be significantly higher in the trauma related group (NES) than in the Elective Care group, indicating that the fracture patients had a significantly higher risk of subsequent fracture than the Elective Care patients. (**B**): FRAX^®^ Score and age by Aetiology. The FRAX score and thus the fracture risk increases continuously with age in both groups. (**C**): FRAX^®^ Score by Age Groups and Aetiology: A comparison of the age groups also shows a higher fracture risk in the trauma group. (***)-*p*-value < 0.001.

When the raw FRAX scores are compared, the NES group showed a significantly higher average fracture risk of 28.1 ± 16.3% compared with 14.6 ± 9.7% in the ES cohort (Figure 1A).

When the FRAX score is related to the age of the patients, a linear regression analysis shows that in both groups the fracture risk after FRAX increases significantly with increasing age (*p* ≤ 0.01). The coefficient of determination was R2 = 0.38 for the NES group and R2 = 0.25 for the elective patients (Figure 1B).

The divisions into the four age groups selected as described above also clearly showed increasing fracture risk with old age (Figure 1C).

### 3.4. Osteoporosis Therapy Information

In the preventive measures we investigated to avoid osteoporosis, the two groups differed significantly in their regular intake of vitamin D. The difference between the two groups was not significant. (NES: 21% and ES: 40%, *p* < 0.001). The proportion of patients with known osteoporosis and specific therapy was almost the same in both groups (Table 1).

### 3.5. Threadiness

80% of elective care patients considered osteoporosis to be a threatening disease for them, whereas the proportion in the NES was significantly smaller at 53.4% (*p* ≤ 0.0001).

### 3.6. Patients’ Osteoporosis Awareness

**Figure 2:**
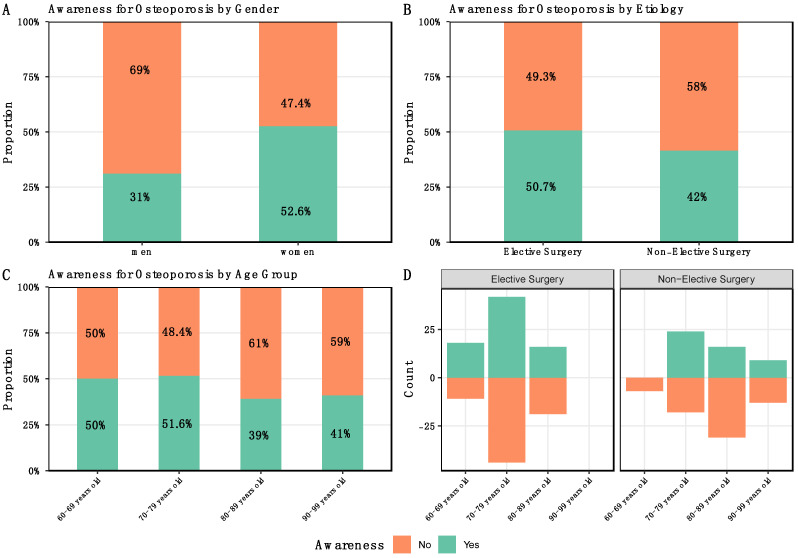
(**A**): Patient’s awareness: (**A**): Patients’ percentage of awareness of osteoporosis by gender, shows an alarming lower awareness among men; (**B**): Patients’ awareness for osteoporosis divided by electivity in percent, among patients not affected by a fracture, a higher percentage has awareness; (**C**): Percentages of patients’ awareness of osteoporosis by age groups; Awareness is highest in the age group 60–69 years and lowest in the age group 80–89 years; (**D**): Patients’ awareness for osteoporosis divided by Electivity and age groups in percent. Only in the 70–79 age group is awareness higher among trauma patients than among elective patients. In the age group 90+ no elective patients could be included.

In accordance with the questionnaire, elective patients had a greater awareness rate of 50.7% compared to individuals who had fractures at 42% (Figure 2B).

Regarding the gender of the patients, it was found that the awareness was significantly higher in women with 52.6% (102/194) than in men with 31.0% (23/74) (*p* ≤ 0.01) (Figure 2A).

It was discovered that awareness of osteoporosis (AO) was highest in those aged 70 to 79, with 51.6%, and lowest in those aged 90 to 99, with 41.0% (Figure 2C).

When the patients are divided into age groups and electivity, it can be seen that in the 60–69 years age group, the ES had 62.0% (*n* = 29) awareness, whereas in the NES group, none of the patients were aware 0% (*n* = 7). In the 70–79 age group, NES patients showed slightly higher awareness (57.0%; *n* = 42) than the ES group (48.8%; *n* = 86). In contrast, in the 80–89 age group, the awareness of ES patients was considerably higher (45.7%; *n* = 45) than that of NES patients (34.0%; *n* = 47).

In the 90+ age group, only patients in the NES group could be included, as elective hip replacement is generally no longer performed in this cohort due to high surgical risk. In the NES group, the proportion of patients with awareness was 41.0% (*n* = 22) (Figure 2D).

The multiple linear regression performed to determine the factors influencing the presence of awareness showed that age, BMI, ASA score, and number of risk factors had no significant influence (Supplementary Table S1). In contrast, patients with female gender had an odds ratio of 1.94 (95% CI: 1.06, −3.64) for the presence of awareness (*p* = 0.035). Patients with no history of fracture had an odds ratio of 0.47 (95% CI: 0.23–0.94) versus those with a positive history of fracture (*p* = 0.033). The ethnology “non-elective” showed a barely non-significant difference with a *p*-value of 0.059 and an odds ratio of 0.54 (95% CI: 0.28–1.02) compared to elective patients.

## 4. Discussion

Our study’s findings demonstrated that patients having elective hip surgery were much more aware of osteoporosis and more likely to receive the right treatment than patients having surgery for an osteoporotic proximal femur fracture.

Awareness of underlying osteoporosis in older trauma patients has a relevant influence on compliance for further diagnosis and treatment [17]. It is evident that the documented reduction in fracture risk of up to 70% cannot be realized in patients, that are not treatment-adherent, because the compliance of oral bisphosphonates is as low as 50% one year after start of therapy. [18,19]. Even if there was an existing fragility fracture or a known risk for further fractures, patients’ willingness to participate in other screening programs (colonoscopy and check-ups) was higher in both groups of patients than in those screening programs for osteoporosis. This reduced awareness is one factor that may help to explain why therapy for osteoporosis is so low in elective and non-elective patients [7]. To frame the results, it is important to keep in mind that in Germany the costs for screening programs for mammography and colonoscopy are covered by the public health insurance. This is currently not the case for osteoporosis screening, which is a major factor for a reduced utilization of osteoporosis screening measures in the general population. In our studies, the costs for the screening examinations were covered by the treating clinics, which meant that the patients did not incur any direct costs for participating in the examinations and thus the cost factor is unlikely to have had any influence on the willingness to participate.

For the best possible surgical management, it is essential to know about the existence of osteoporosis or osteopenia. This is because the stability of the bone already plays an important role in the selection of the prosthesis and its application. Patients undergoing hip arthroplasty today are the patients who are at risk of suffering periprosthetic femur fracture in the future. Therefore, the knowledge of bone health is crucial for selecting a suitable drug therapy that positively influences implant osseointegration and reduces the risk of periprosthetic fracture [20,21].

The fracture risk determined by means of FRAX is clearly correlated with age in our patients, since on the one hand age is included in the score and on the other hand with increasing age basically more comorbidities are to be expected, which are also recorded in the score Given that a fracture in the past is considered a risk factor in the FRAX score, it makes sense that individuals who have recently undergone trauma have a higher fracture risk. However, this increased risk in the NES group is not associated with increased awareness compared to elective patients. The reason for this is multifactorial. First, it has been shown that fracture patients are generally less aware of preventive examinations (NES: 67%; ES: 85%), suggesting reduced health awareness in this group. Second, the fracture patients presumably have fewer physician contacts due to musculoskeletal problems than patients who receive a hip prosthesis due to chronic arthritic disease of the corresponding joint. Due to this mostly long-standing disease, the physicians have more opportunities to create a higher awareness of the disease with regard to the musculoskeletal system during the treatments than with the fracture patients who are acutely admitted to the clinic. Furthermore, elective patients rate their health status during hospitalization significantly lower than non-elective patients, indicating a higher level of suffering among elective patients than among acute patients, which makes them more likely to feel threatened by illness than the supposedly healthier fracture patients. The ASA score collected anesthesiologists, contradicts the subjective assessment of the patients, as it rates the fracture patients as more severely affected than the elective patients. Another relevant factor is the fact that the patients who were acutely hospitalized after a fall consider chronic diseases to be less threatening than patients who are already chronically ill with arthrosis. In addition, on the surgical side, the focus is on the best possible treatment of the acute injury and less on the detection of any additional osteoporosis that may be present. This can lead to a communication and perception problem in fracture patients, as they attribute their suffering to the acute trauma and are not aware of the risk of a further fragility fracture. Patients frequently may not understand the order of causation, believing that the damage was first caused by diminished bone stability rather than the actual fall. Accordingly, physicians should focus on creating the best possible understanding of the disease and the best possible awareness among patients. This is a cornerstone for an early start of therapy, good therapy compliance and adherence, which is essential for successful subsequent fracture prophylaxis. This is particularly important for secondary prevention in fracture patients, but also relevant for primary prevention in elective patients. Mortality after PFF has remained almost the same over the last 30 years, still significantly increased in the first year, up to 21–24% depending on the literature [22].

Therefore, Lyles et al. were able to demonstrate another important argument for the postoperative implementation of specific therapy for osteoporosis after PFF: iv administration of 5 mg zoledronic acid within 90 days postoperatively significantly reduced mortality by 28% in this group [23].

Another important point for primary as well as secondary prevention is a regular intake of vitamin D. Among the elective patients before surgery, twice as much vitamin D was substituted as among the NES patients. The reasons for this could be a possibly increased health awareness among the ES patients combined with a higher awareness of osteoporosis among them. Presumably, ES patients generally pay more attention to a healthy lifestyle and are therefore more likely to take vitamin D regularly as a preventive measure.

Similar to this, after coronary stent placement, chronic medication and close monitoring are much more frequently used in internal medicine to manage disorders, such as hypertension and hypercholesterolemia, which cause artery narrowing and calcification. Additionally, the health care system’s diverse systems frequently make consistent therapy challenging. Models such as the Fracture Liaison Service have been developed to monitor patients over the long term, including via many healthcare system sectors, to help with this [24,25,26].

It is important to take into account the study’s limitations, such as the fact that it was limited to one university hospital and one affiliated hospital. A multicenter research would be really intriguing to perform. Although patients with cognitive impairment were not included in the study, it is feasible that patients’ understanding of the underlying osteoporosis may differ depending on their degree of education, even though this study did not assess educational level.

To our knowledge, the current study is the first to investigate whether patients undergoing elective and nonelective hip surgery are aware of the underlying osteoporosis.

## 5. Conclusions

We demonstrated that in all groups, willingness to participate in other screening programs (preventive examinations and colonoscopy) was significantly higher than willingness to participate in further osteoporosis diagnostics. Therefore, implementation of a screening and care program for osteoporosis such as Fracture Liaison Services (FLS) may improve patient awareness of this condition especially among fracture patients.

## Figures and Tables

**Table 1:** Demographic data and characteristics of the study population; Body Mass Index (BMI); Physical Status Classification System (ASA); Dachverband für Osteologie (DVO). * statistically significant *p* ≤ 0.05, ** statistically significant *p* ≤ 0.01, *** statistically significant *p* ≤ 0.001.

Results	Non-Elective Surgery	Elective Surgery	*p*-Value	Significance Level
	*n* = 118	*n* = 150		
Age [years (±SD)]	81.25 (±7.80)	75.0 (±6.40)	<0.001	***
G1 (60–70 years)	10 (8.5%)	31 (20.7%)	0.009	**
G2 (71–79 years)	43 (36.4%)	89 (59.3%)	<0.001	***
G3 (80–90 years)	62 (41%)	30 (20.0%)	<0.001	***
G4 (91–100 years)	13 (11%)	0 (0.0%)	---	---
Gender				
male	25 (21%)	49 (33%)	0.037	*
female	93 (79%)	101 (67%)	0.037	*
BMI [kg/m^2^ (±SD)]	24.05 (±4.27)	25.12 (±3.94)	0.031	*
<20 kg/m^2^	10 (8.5%)	6 (4.0%)	0.016	*
20–24.9 kg/m^2^	66 (55.9%)	65 (43.3%)	0.054	ns
25–29.9 kg/m^2^	30 (25.4%)	66 (44.0%)	0.002	**
30–34.9 kg/m^2^	10 (8.5%)	11 (7.33)	0.907	ns
≥35 kg/m^2^	2 (1.7%)	2 (1.33)	1.000	ns
ASA Score [Score; (±SD)]	2.61 (±0.58)	2.05 (±0.49)	<0.001	***
1	2 (1.7%)	13 (8.7%)	0.028	*
2	45 (38.1%)	117 (71.0%)	<0.001	***
3	67 (56.8%)	19 (12.7)	<0.001	***
4	4 (3.4%)	1 (0.7%)	0.237	ns
Risk Factors (DVO) [*n*; (%)]				
Parental hip fracture	37 (31.0%)	27 (18.0%)	0.016	*
Current smoker	12 (10.0%)	10 (7.0%)	0.416	ns
Glucocorticoid use	16 (14.0%)	20 (13.0%)	1.000	ns
Rheumatoid arthritis	6 (5.0%)	8 (5.0%)	1.000	ns
Menopause < 45 year	31 (26.3%)	44 (29.3%)	0.676	ns
Aromatase inhibitor use	9 (8.0%)	1 (1.0%)	0.007	**
Alcohol (>1 bottle of beer a day)	16 (14.0%)	21 (14.0%)	0.701	ns
No regular consumption of dairy products	35 (30.0%)	24 (16.0%)	0.011	*
Cumulative number of risk factors per patient	1.86 (±1.45)	1.25 (±1.12)	<0.001	***
Osteoporosis Therapy Information [*n*; (%)]				
Regular vitamin D intake	25 (21.0%)	60 (40.0%)	0.002	**
Known osteoporosis diagnosis	29 (25.0%)	41 (27.0%)	0.711	ns
Osteoporosis medication intake	27 (23.0%)	37 (25.0%)	0.845	ns
Patient Reported Health Status (1–10)	4.46 (±2.42)	3.87 (±2.42)	0.048	*

## Data Availability

The corresponding author can provide the data described in this study upon request. Due to laws governing data protection and privacy, the data are not accessible to the general public.

## Supplementary Materials

The following are available online at https://www.mdpi.com/article/10.3390/medicina58111564/s1, Table S1: Results of multiple linear regression performed to determine the factors influencing the presence of awareness for osteoporosis.

## References

[1] Kanis J.A., Odén A., McCloskey E.V., Johansson H., Wahl D.A., Cooper C. (2012). A Systematic Review of Hip Fracture Incidence and Probability of Fracture Worldwide. Osteoporos. Int..

[2] Hagen G., Magnussen J., Tell G., Omsland T. (2020). Estimating the Future Burden of Hip Fractures in Norway. A NOREPOS Study. Bone.

[3] Downey C., Kelly M., Quinlan J.F. (2019). Changing Trends in the Mortality Rate at 1-Year Post Hip Fracture—A Systematic Review. World J. Orthop..

[4] Dyer S.M., Crotty M., Fairhall N., Magaziner J., Beaupre L.A., Cameron I.D., Sherrington C. (2016). A Critical Review of the Long-Term Disability Outcomes Following Hip Fracture. BMC Geriatr..

[5] Desai R.J., Mahesri M., Abdia Y., Barberio J., Tong A., Zhang D., Mavros P., Kim S.C., Franklin J.M. (2018). Association of Osteoporosis Medication Use After Hip Fracture With Prevention of Subsequent Nonvertebral Fractures: An Instrumental Variable Analysis. JAMA Netw. Open.

[6] Keppler A.M., Kraus M., Blaschke M., Thomasser N., Kammerlander C., Böcker W., Neuerburg C., Stumpf U.C. (2021). Reduced Awareness for Osteoporosis in Distal Radius Fracture Patients Compared to Patients with Proximal Femur Fractures. J. Clin. Med..

[7] Bernatz J.T., Brooks A.E., Squire M.W., Illgen R.I., Binkley N.C., Anderson P.A. (2019). Osteoporosis Is Common and Undertreated Prior to Total Joint Arthroplasty. J. Arthroplast..

[8] Bernatz J.T., Krueger D.C., Squire M.W., Illgen R.L., Binkley N.C., Anderson P.A. (2019). Unrecognized Osteoporosis Is Common in Patients With a Well-Functioning Total Knee Arthroplasty. J. Arthroplast..

[9] Setty N., LeBoff M.S., Thornhill T.S., Rinaldi G., Glowacki J. (2011). Underestimated Fracture Probability in Patients With Unilateral Hip Osteoarthritis as Calculated by FRAX. J. Clin. Densitom..

[10] Puth M.-T., Klaschik M., Schmid M., Weckbecker K., Münster E. (2018). Prevalence and Comorbidity of Osteoporosis—A Cross-Sectional Analysis on 10,660 Adults Aged 50 Years and Older in Germany. BMC Musculoskelet. Disord..

[11] Notarnicola A., Maccagnano G., Tafuri S., Moretti L., Laviola L., Moretti B. (2016). Epidemiology of Diabetes Mellitus in the Fragility Fracture Population of a Region of Southern Italy. J. Biol. Regul. Homeost. Agents.

[12] Notarnicola A., Tafuri S., Maccagnano G., Moretti L., Moretti B. (2017). Frequency of Hypertension in Hospitalized Population with Osteoporotic Fractures: Epidemiological Retrospective Analysis of Hospital Discharge Data in the Apulian Database for the Period 2006–2010. Eur. J. Inflamm..

[13] Ciatti C., Maniscalco P., Quattrini F., Gattoni S., Magro A., Capelli P., Banchini F., Fiazza C., Pavone V., Pagliarello C.P. (2021). The Epidemiology of Proximal Femur Fractures during Covid-19 Emergency in Italy: A Multicentric Study. Acta Biomed..

[14] Dachverband Osteologie e.V. Prophylaxe, Diagnostik und Therapie der OSTEOPOROSE. https://www.dv-osteologie.org/uploads/Leitlinie2017/FinaleVersionLeitlinieOsteoporose2017_end.pdf.

[15] Kanis J.A., Harvey N.C., McCloskey E., Bruyère O., Veronese N., Lorentzon M., Cooper C., Rizzoli R., Adib G., Al-Daghri N. (2020). Algorithm for the Management of Patients at Low, High and Very High Risk of Osteoporotic Fractures. Osteoporos. Int..

[16] Neuerburg C., Mittlmeier L., Schmidmaier R., Kammerlander C., Böcker W., Mutschler W., Stumpf U. (2017). Investigation and Management of Osteoporosis in Aged Trauma Patients: A Treatment Algorithm Adapted to the German Guidelines for Osteoporosis. J. Orthop. Surg. Res..

[17] Boudreau D.M., Yu O., Balasubramanian A., Wirtz H., Grauer A., Crittenden D.B., Scholes D. (2017). A Survey of Women’s Awareness of and Reasons for Lack of Postfracture Osteoporotic Care. J. Am. Geriatr. Soc..

[18] Ziller V., Kostev K., Kyvernitakis I., Boeckhoff J., Hadji P. (2012). Persistence and Compliance of Medications Used in the Treatment of Osteoporosis—Analysis Using a Large Scale, Representative, Longitudinal German Database. Int. J. Clin. Pharmacol. Ther..

[19] Murad M.H., Drake M.T., Mullan R.J., Mauck K.F., Stuart L.M., Lane M.A., Abu Elnour N.O., Erwin P.J., Hazem A., Puhan M.A. (2012). Comparative Effectiveness of Drug Treatments to Prevent Fragility Fractures: A Systematic Review and Network Meta-Analysis. J. Clin. Endocrinol. Metab..

[20] Karachalios T.S., Koutalos A.A., Komnos G.A. (2020). Total Hip Arthroplasty in Patients with Osteoporosis. HIP Int..

[21] Russell L.A. (2013). Osteoporosis and Orthopedic Surgery: Effect of Bone Health on Total Joint Arthroplasty Outcome. Curr. Rheumatol. Rep..

[22] Mundi S., Pindiprolu B., Simunovic N., Bhandari M. (2014). Similar Mortality Rates in Hip Fracture Patients over the Past 31 Years. Acta Orthop..

[23] Lyles K.W., Colón-Emeric C.S., Magaziner J.S., Adachi J.D., Pieper C.F., Mautalen C., Hyldstrup L., Recknor C., Nordsletten L., Moore K.A. (2007). Zoledronic Acid and Clinical Fractures and Mortality after Hip Fracture. N. Engl. J. Med..

[24] Åkesson K.E., McGuigan F.E.A. (2021). Closing the Osteoporosis Care Gap. Curr. Osteoporos. Rep..

[25] Gosch M., Kammerlander C., Neuerburg C. (2019). Osteoporosis—Epidemiology and Quality of Care. Z. Gerontol. Geriatr..

[26] Geiger I., Kammerlander C., Höfer C., Volland R., Trinemeier J., Henschelchen M., Friess T., Böcker W., Sundmacher L., FLS-CARE study group (2021). Implementation of an Integrated Care Programme to Avoid Fragility Fractures of the Hip in Older Adults in 18 Bavarian Hospitals—Study Protocol for the Cluster-Randomised Controlled Fracture Liaison Service FLS-CARE. BMC Geriatr..

