# Supplementary material for: Reduced Awareness for Osteoporosis in Hip Fracture Patients Compared to Elderly Patients Undergoing Elective Hip Replacement
Source: Medicina (Kaunas). 2022 Oct 31;58(11):1564. doi: 10.3390/medicina58111564 (PMC9696469; doi:10.3390/medicina58111564)
Supplement: Supplementary file 1 [file medicina-58-01564-s001.zip › medicina-1941409-supplementary.pdf]

Supplementary:

Table S1: Results table of multiple linear regression for factors influencing awareness of osteoporosis.

**Characteristic**	**OR**[1]	**95% CI**[1]	***p*-value**
Age	0.98	0.95, 1.02	0.4
Gender			
Men	—	—	
Women	1.94	1.06, 3.64	0.035
BMI	0.97	0.91, 1.03	0.3
ASA-Score	0.85	0.51, 1.39	0.5
Etiology			
Elective Surgery	—	—	
Non-elective Surgery	0.54	0.28, 1.02	0.059
Number of risk factors	1.19	0.94, 1.50	0.15
Previous fracture			
Yes	—	—	
No fracture	0.47	0.23, 0.94	0.033

[1] OR = Odds Ratio, CI = Confidence Interval
